# PPAR*γ* regulated CIDEA affects pro-apoptotic responses in glioblastoma

**DOI:** 10.1038/cddiscovery.2015.38

**Published:** 2015-11-23

**Authors:** A Chatterjee, P Mondal, S Ghosh, VS Mehta, E Sen

**Affiliations:** 1 Cellular and Molecular Neuroscience Division, National Brain Research Centre , Haryana, India; 2 Paras Hospitals, Gurgaon, India

## Abstract

Refractoriness of glioblastoma multiforme (GBM) to current treatment paradigms has necessitated identification of new targets to better the existing therapeutic strategies. One such target is peroxisome proliferator-activated receptor gamma (PPAR*γ*) – a transcription factor involved in regulation of lipid metabolism and inflammation. Expression of PPAR*γ*, a known regulator of cell death-inducing DFFA-like effector (CIDEA), is modulated by hypoxia inducible factor (HIF-1*α*). While the involvement of CIDEA in lipid metabolism is known, its role in malignancies remains largely unknown. An elevated PPAR*γ* and low CIDEA level was observed in GBM tumors as compared with surrounding non-neoplastic tissue. As reciprocal relation exists between PPAR and HIF-1*α*: and as HIF-1*α* is a key component in glioma progression, their role in regulating CIDEA expression in glioblastoma was investigated. Although HIF-1*α* inhibition had no effect on CIDEA expression, pharmacological inhibition of PPAR*γ* elevated CIDEA levels. PPAR*γ* mediated upregulation of CIDEA was accompanied by decreased recruitment of NF*κ*B and SP1 to their predicted binding sites on CIDEA promoter. Ectopic expression of CIDEA triggered apoptosis, activated JNK, decreased HIF-1*α* activation and increased PPAR*γ* levels in glioma cells. While CIDEA overexpression induced actin cytoskeletal disruption, cell cycle arrest, release of pro-inflammatory cytokine IL-6 in a JNK-dependent manner; CIDEA mediated apoptotic cell death, decreased STAT3 phosphorylation and increased p53 acetylation was JNK independent. This study highlights for the first time the existence of (i) PPAR*γ*-CIDEA regulatory loop in glioma and (ii) novel function of CIDEA as regulator of glioma cell survival.

## Introduction

As dysregulated metabolism promotes malignancy, targeting the regulatory genes of metabolic pathways is emerging as a viable therapeutic approach. Among the three members of cell death-inducing DFFA-like effector (CIDE) protein family, the role of CIDEA in lipid metabolism is documented.^1^ CIDEA is also known to induce apoptosis.^2^ Though a low expression level of CIDEA in different malignancies including glioblastoma multiforme (GBM) is documented,^3^ the mechanisms of its regulation and role in tumor progression remain unexplored.

Proliferator-activated receptor (PPAR) is an important positive regulator of CIDEA expression in murine liver,^4^ and PPAR*γ* is a hypoxia inducible factor (HIF1*α*) target. As HIF-1*α* is associated with poor prognosis in glioblastoma, targeting the HIF pathway is considered as an important therapeutic strategy.^5–7^ Interestingly, evidences suggest mutual inhibitory mechanisms between PPAR and HIF-1*α*. Although hypoxic stress-induced HIF-1*α* negatively regulates PPAR*γ* during adipocytic differentiation,^8^ pathologic stress-induced PPAR*γ* upregulation involves HIF-1*α* activation in cardiomyocytes.^9^ Besides, PPAR*γ* agonist pioglitazone has been reported to inhibit glioma survival.^10^ Given the importance of HIF-1*α* in glioma biology, and involvement of PPAR*γ* in CIDEA expression; we investigated the (i) role of HIF-1*α* and PPAR*γ* in CIDEA expression and (ii) the role of CIDEA in glioma cell survival.

## Results

### Elevated PPAR*γ* and low CIDEA levels in GBM tumors

**Figure 1:**
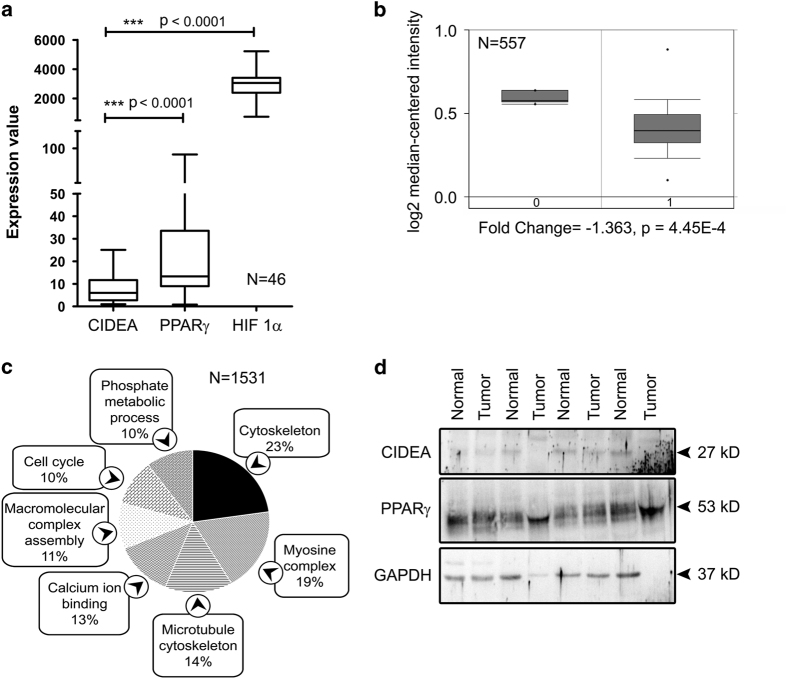
PPAR*γ* and CIDEA expression in glioblastoma. (**a**) CIDEA expression is significantly low in GBM tumors from different regions of brain as indicated by Gene Expression Omnibus (GEO) database (data set record no. GDS4470). The significance is calculated by two-tailed Mann–Whitney *U*-test. (**b**) CIDEA mRNA expression is significantly low in GBM as compared with normal brain tissue. Figure is presented of TCGA expression profiles as retrieved from Oncomine database. (**c**) Significantly enriched pathways from co-expressed genes of CIDEA. Co-expression data were collected from Oncomine with >80% correlation coefficient in GBM and analyzed using DAVID Bioinformatics Resource 6.7. (**d**) Western blot analysis demonstrating levels of CIDEA and PPAR*γ* in GBM tumor as compared with surrounding non-neoplastic tissue. The figure shows blots from four independent tumor samples. Blot was re-probed for GAPDH to establish equal loading.

CIDEA mRNA expression was found to be significantly lower than PPAR*γ* and HIF-1*α* in 46 GBM tissue samples from different regions of brain (Gene Expression Omnibus (GEO) data set number: GDS4470, Figure 1a). In addition, a significantly low expression of CIDEA mRNA was also observed in GBM tumors as compared with normal brain tissue (oncomine, TCGA mRNA expression data, Figure 1b). Genes that are tightly co-expressed with CIDEA in glioblastoma were found to be enriched in cytoskeleton pathways (enrichment score: 23%), myosin complex assembly (19%), microtubule functioning (14%), calcium-ion binding (13%), macromolecular complex assembly (11%), cell cycle (10%) and phosphate metabolic process (10%) (Figure 1c). Western blot analysis also revealed an almost undetectable level of CIDEA in GBM tumor samples as well in surrounding normal tissues. This low expression of CIDEA was concomitant with elevated PPAR*γ* levels observed in GBM tumors as compared with the surrounding non-neoplastic tissue (Figure 1d).

### PPAR*γ* regulates CIDEA expression but HIF-1*α* has no role

**Figure 2:**
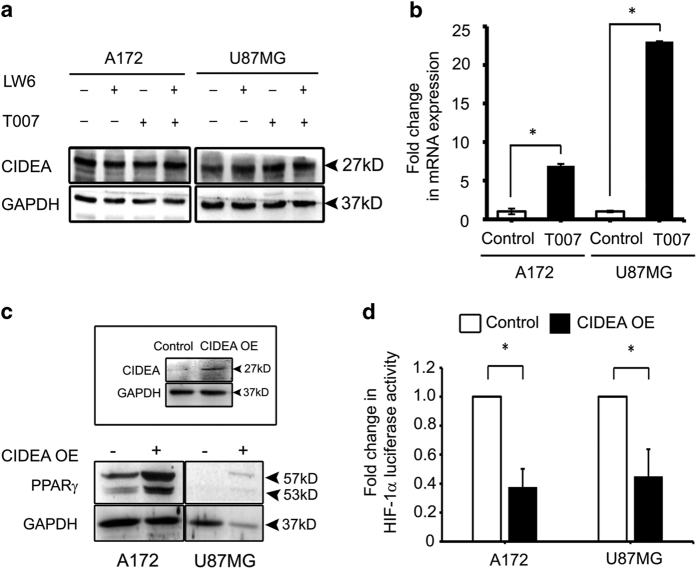
Inter-regulatory relationship between PPAR*γ* and CIDEA. (**a**) Western blot showing effect of PPAR*γ* and/or HIF-1*α* inhibition on CIDEA protein expression in glioma cell lines. Blots were re-probed for GAPDH to establish equivalent loading. (**b**) Real-time PCR indicating elevated CIDEA mRNA expression on PPAR*γ* inhibition. Graph represents fold change of CIDEA total mRNA expression. 18s rRNA was used as control. (**c**) Ectopic expression of CIDEA elevates PPAR*γ* levels in glioma cell lines. Inset showing heightened CIDEA on transfection with overexpression (OE) construct. Blots were re-probed for GAPDH to established equal loading. (**a** and **c**) Representative blot from three independent experiments with identical results. (**d**) CIDEA negatively regulates HIF-1*α* transcriptional activation. The graphs represent fold change in HIF-1*α* luciferase reporter activity over control in cells transfected with CIDEA overexpression construct. Values (**b** and **d**) represent the means±S.E.M. from three independent experiments. * denotes significant change from control (*P*<0.05). LW6 and T007 are HIF-1*α* and PPAR*γ* inhibitors, respectively.

HIF-1*α* and PPAR*γ* are known to negatively regulate each other.^11^ As PPAR*γ* is known to regulate CIDEA, and as HIF-1*α* is a potential antiglioma target;^6^ the involvement of HIF-1*α* and PPAR*γ* in regulation of CIDEA expression in glioma cells was investigated. Glioma cells were treated with LW6 and/or T0070907 either alone or in combination. LW6 inhibits HIF-1*α* accumulation and suppresses the expression of hypoxia-induced genes,^12^ and PPAR*γ* antagonist T0070907 inhibits activation of PPAR*γ*.^13^ Inhibition of PPAR*γ* elevated CIDEA expression in glioma cells (Figure 2a). However, treatment with HIF-1*α* had no effect on CIDEA level (Figure 2a). The increased CIDEA levels observed on PPAR*γ* inhibition remained unaffected on co-treatment with HIF-1*α* inhibitor (Figure 2a). These findings suggested that CIDEA expression in glioma cells is independent of HIF-1*α* but PPAR*γ* dependent. As PPAR*γ* affected CIDEA protein expression, we determined CIDEA mRNA expression on PPAR*γ* inhibition (Figure 2b). Inhibition of PPAR*γ* increased CIDEA mRNA expression significantly (Figure 2b). The extent of increase in mRNA levels in A172 and U87MG corresponded to the changes in protein expression observed in these two cell lines upon PPAR*γ* inhibition.

### CIDEA overexpression increases PPAR*γ* expression and decreases HIF-1*α* activation

We next investigated the consequences of elevated CIDEA expression on HIF-1*α* and PPAR*γ* expression. This was accomplished by transfecting cells with CIDEA overexpression construct. Increase in CIDEA levels was concomitant with increase in PPAR*γ* expression, albeit to different extent in different cell lines (Figure 2c). This suggested the existence of a PPAR*γ*- CIDEA regulatory loop. Though HIF-1*α* inhibition had no effect of CIDEA expression, overexpression of CIDEA decreased HIF-1*α* transcriptional activation (Figure 2d). Thus, CIDEA negatively regulates HIF-1*α* activation in glioma cells.

### PPAR*γ* inhibition decreases recruitment of SP1 and NF*κ*B on CIDEA promoter

**Figure 3:**
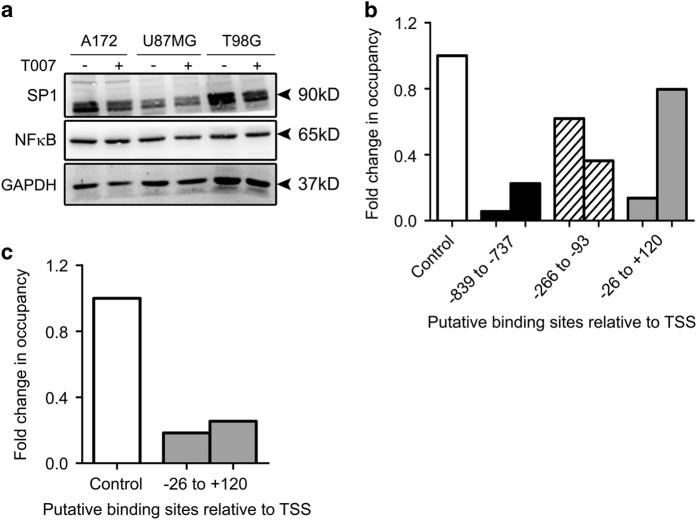
Inhibition of PPAR*γ* reduces NF*κ*B and SP1 binding on CIDEA promoter. (**a**) PPAR*γ* inhibitior T007 has no effect on NF*κ*B and SP1 expression. Blot is representative of two independent experiments. Blots were re-probed for GAPDH to establish equal loading. (**b**) ChIP-qPCR assays demonstrating decreased binding of NF*κ*B to its cognate sites on CIDEA promoter. DNA isolated from control and PPAR*γ* inhibitor treated A172 glioma cells pre and post immunoprecipitation with anti-NF*κ*B antibody, was amplified using specific primer sets. Binding affinity of NF*κ*B was found to be low at three different putative binding sites on CIDEA promoter on inhibition of PPAR*γ*. (**c**) PPAR*γ* inhibition decreases SP1 binding to its cognate site on CIDEA promoter at −26 to +120 position, as indicated by ChIP-qPCR assay. Graph (**b** and **c**) represents fold change as calculated from Ct values of two independent experiments for a single site.

Constitutively activated NF*κ*B in GBM tumors promotes their growth and survival.^14^ PPAR*γ* is known to induce proteosomal degradation of NF*κ*B,^15^ and NF*κ*B regulates CIDEA expression.^16^ As there was no significant change in expression of NF*κ*B upon PPAR*γ* inhibition (Figure 3a), we determined whether PPAR*γ* inhibition affects DNA-binding pattern of NF*κ*B on CIDEA promoter. To study the mechanistic detail of PPAR*γ* mediated transcriptional regulation of CIDEA in glioma cell, we chose a 1120 bp genomic region (−1000 to +120) on the CIDEA promoter. All the base pair positions mentioned here are according to the TSS position described by Petterson *et al.* ^16^ NF*κ*B-binding sites were located at −839 to −737, −266 to −93 and −26 to +120 regions. Chromatin immunoprecipitation (ChIP) real-time PCR revealed an overall decrease in recruitment of NF*κ*B to these sites on inhibition of PPAR*γ*, as compared with control (Figure 3b). Elevated SP1 levels has been suggested as a prognostic marker in gliomas^17^ and a strong association of SP1 with NF*κ*B is involved in transcriptional regulation of genes.^18^ As *in silico* analysis revealed a SP1 binding site at −26 to +120 on the CIDEA promoter containing the NF*κ*B site, the effect of PPAR*γ* inhibition on SP1 expression and binding to this site on CIDEA promoter was also investigated. While inhibition of PPAR*γ* had no effect on SP1 levels, a decrease in SP1 binding to the CIDEA promoter was observed (Figure 3c). Thus, the decreased recruitment of NF*κ*B to its putative binding sites on CIDEA promoter observed upon PPAR*γ* inhibition was accompanied by a low enrichment of SP1 to its cognate site overlapping the NF*κ*B site.

### Role of JNK in CIDEA mediated cell cycle arrest and death

**Figure 4:**
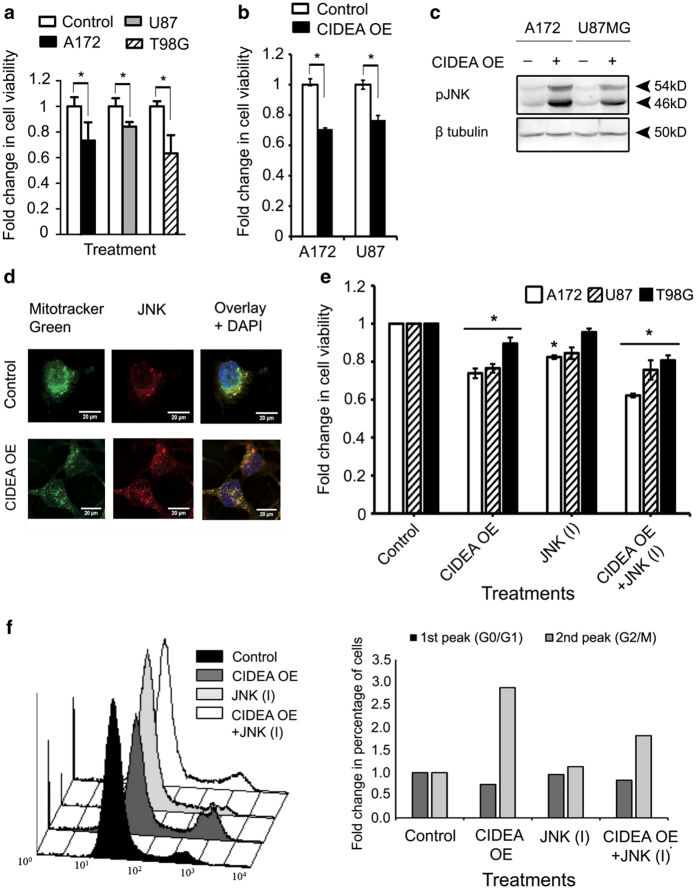
CIDEA overexpression induces cell death. (**a**) PPAR*γ* inhibition reduces glioma cell viability. The graph represents the viable cells, fold change over control, observed when glioma cells were treated with 50 *μ*M of T0070907 for 40 h, as determined by MTS assay. (**b**) CIDEA overexpression reduces cell viability in glioma cell lines, as determined by MTS. (**c**) CIDEA overexpression induces phospho-JNK expression in glioma cells. Blot is representative of three independent experiments. Blots were re-probed for *β*-tubulin to establish equal loading control. (**d**) CIDEA overexpression induces JNK co-localization into the mitochondria. Representative image showing mitochondria stained with mitotracker green, JNK with Alexa Fluor 594-tagged secondary antibody (red) and nucleus with DAPI (blue). (**e**) CIDEA-mediated glioma cell death is JNK independent. Graph represents the viable cells, fold change over control, observed when CIDEA overexpressing cells were treated with 10*μ*M JNK inhibitor for 40 h, as measured by MTS assay. The graph (**a**, **b**, **e**) represents the means±S.E.M. from three independent experiments. * Significant decrease from control (*P*≤0.05). (**f**) Overexpression of CIDEA induces JNK-mediated G2/M phase cell cycle arrest in A172 glioma cell line. Left panel shows population of cells arrested at G2/M phase, the right panel indicates the fold change in cell population. Graph is representative data of two independent experiments.

As PPAR*γ* inhibition-induced CIDEA was accompanied by decrease in glioma cell viability (Figure 4a), we next investigated the consequences of CIDEA overexpression on glioma cell survival. Ectopic expression of CIDEA induced cell death (Figure 4b) was concomitant with induction of JNK phosphorylation (Figure 4c). Increased localization of JNK to the mitochondria was observed upon CIDEA overexpression (Figure 4d). As JNK has been implicated as an inducer of apoptosis in glioma cells,^19,20^ and since JNK mediated apoptotic cell death involves its mitochondrial localization,^21^ the role of JNK in CIDEA mediated cell death was investigated. However, JNK inhibition failed to rescue CIDEA-mediated glioma cell death (Figure 4e). Although CIDEA-mediated induction of Caspase-3 activation was found to be independent of JNK (Supplementary Figure 1), G_2_/M phase cell cycle arrest was found to be JNK dependent (Figure 4f). However, the expression of cyclin B1 associated with G2/M phase of cell cycle arrest was elevated only in A172 cells (Supplementary Figure 2). Thus, CIDEA-mediated cell death is JNK independent but cell cycle arrest is JNK mediated.

### CIDEA induces p53 acetylation in glioma cell

**Figure 5:**
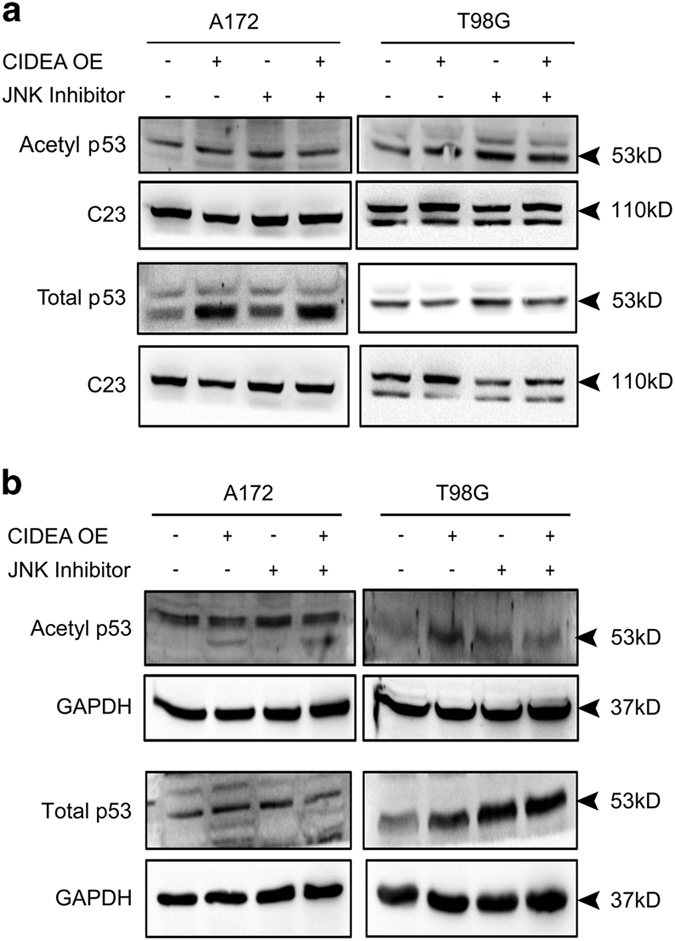
Overexpression of CIDEA increases p53 acetylation in a JNK-independent manner. CIDEA overexpression affects acetylated p53 and total p53 levels in the nucleus (**a**) and the cytosol (**b**) of glioma cells in a JNK-independent manner. A representative blot is shown from three independent experiments with identical results. Blots were re-probed with C23 (for nuclear extract) or GAPDH (for cytosolic extract) as loading control.

As ectopic expression of CIDEA induced cell death, we investigated the status of p53 in CIDEA overexpressing cells. CIDEA induced p53 expression in a JNK-independent manner both in wild-type and mutant p53 glioma cells. Interestingly, p53 was localized in the nucleus of p53 wild-type A172 cells (Figure 5a), whereas a cytosolic localization was observed in p53 mutant cells T98G (Figure 5b). As acetylation of p53 increases its transcriptional activity as well as the transcription independent pro-apoptotic function,^22^ the status of acetylated p53 in CIDEA overexpressing cells was investigated. Increased acetylation of p53 in cell lines containing transcriptionally active or inactive p53 was observed on CIDEA overexpression (Figures 5a and b).

### JNK regulates CIDEA mediated disruption of actin cytoskeletal

**Figure 6:**
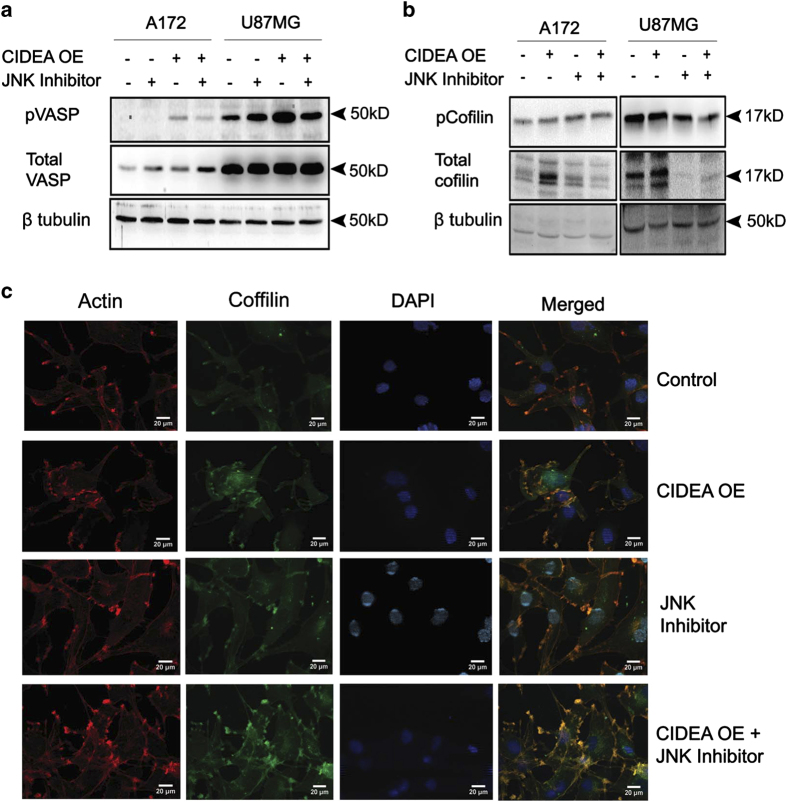
JNK regulates CIDEA mediated actin cytoskeletal disruption. CIDEA overexpression alters the levels of phospho-VASP (**a**) and phospho-Cofilin (**b**) in glioma cell lines in a JNK dependent manner. A representative blot is shown from two independent experiments with identical results. Blots were re-probed with *β*-tubulin to establish loading control. (**c**) CIDEA overexpression affects actin cytoskeleton architecture in a JNK-dependent manner. Confocal imaging depicts that association between Cofilin (green, Alexa Fluor 488) and actin (red, rhodamine-phalloidin) observed in CIDEA overexpressing cells is reverted by JNK inhibition. Representative images from two independent experiments are shown for indicated conditions.

CIDEA overexpression was accompanied by altered cell morphology, suggestive of disrupted cytoskeletal architecture (Supplementary Figure 3). Vasodilator-stimulated phosphoprotein (VASP) signaling is critical for dynamic actin reorganization, and VASP phosphorylation controls actin cytoskeleton remodeling.^23^ Inactivation of cofilin by its phosphorylation leads to accumulation of actin filaments.^24^ The elevated level of active phosphorylated VASP (Figure 6a) and non-phosphorylated Cofilin (Figure 6b) observed in CIDEA overexpressed cell was abrogated upon JNK inhibition. Immuno-cytochemical studies using antibodies directed against cofilin and direct labelling of actin using fluorophore-tagged Rhodamine further suggested that CIDEA over-expression disrupts polymerized F-actin structure in a JNK dependent manner (Figure 6c). Similar co-localization experiment performed in U87 and T98G glioma cells yielded identical results (data not shown).

### CIDEA affects IL-6 and STAT3 activation

**Figure 7:**
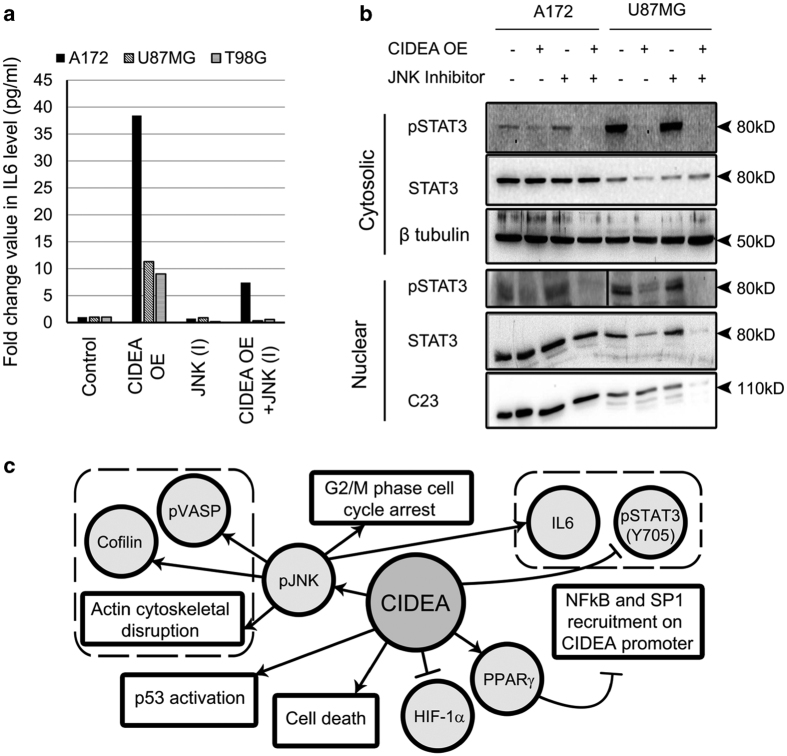
CIDEA induces IL-6 levels but abrogates STAT3 activation in glioma cells. (**a**) Ectopic expression of CIDEA elevates IL-6 secretion in a JNK-dependent manner as revealed by Cytokine Bead Array. Graph represents representative data from two independent experiments in three cell lines. (**b**) CIDEA overexpression abrogates cytosolic and nuclear pSTAT3 (Y705) levels in a JNK independent manner. Blots were re-probed with C23 (for nuclear extract) or *β*-tubulin (for cytosolic extract) to establish equal loading. Blots shown are representative of three independent experiments. (**c**) Proposed mechanism of regulation of CIDEA and its role in glioma cell survival.

PPAR*γ* regulates DNA binding and transactivation of STAT3,^25^ and IL-6 induced STAT3 activation is associated with pro-survival responses in glioma cells.^26^ CIDEA overexpression increased IL-6 levels in a JNK-dependent manner (Figure 7a). Despite elevating IL-6 levels, CIDEA overexpression abrogated STAT3 phosphorylation in a JNK-independent manner (Figure 7b). Taken together, our findings indicate that CIDEA regulates several pathways associated with glioma cell survival (Figure 7c).

## Discussion

There has been opposing reports regarding the ability of PPAR*γ* to effect tumor progression as both agonists and antagonists have demonstrated anti-tumorigenic properties.^27^ PPAR*γ* agonists have shown potential anti-glioma effects.^10^ As low expression of CIDEA in glioma is concomitant with elevated levels of PPAR*γ* and HIF-1*α*, this study was undertaken to understand the (i) role of PPAR*γ* and HIF-1*α* in maintaining the low basal expression of CIDEA in GBM, and (ii) the effect of CIDEA overexpression on glioma cell survival. Our findings suggest that inhibition of PPAR*γ* enhances CIDEA expression, whereas HIF-1*α* inhibition has no effect. Interestingly, increased CIDEA levels triggered glioma cell apoptosis, decreased HIF-1*α* activation and elevated PPAR*γ* levels.

Although promoter of CIDEA in murine liver cell contains PPAR*γ* inducible PPREs associated with CIDEA expression,^4^ human CIDEA promoter sequence lacks PPREs. Moreover, PPAR*γ* agonist has failed to induce CIDEA transcriptional activity in human adipocytic cell.^16^ This suggests that the possibility of direct DNA-binding activity of PPAR*γ* to human CIDEA promoter is negligible. A NF*κ*B site at position −163/−151 serves as an important modulator for TNF-mediated downregulation of CIDEA expression.^16^ Besides, PPAR*γ* can regulate NF*κ*B activation and DNA binding.^15^ Also, SP1 and SP3 binding is crucial for positive regulation of non-methylated CIDEA promoter in human adipocytes.^28^ Our study revealed that PPAR*γ* inhibition affects the recruitment of NF*κ*B and SP1 to their cognate sites on CIDEA promoter. It is possible that increased binding of these two factors on CIDEA promoter in GBM exhibiting elevated NF*κ*B, SP1 and PPAR*γ* levels, could contribute to the low expression of CIDEA in these tumors.

PPAR*γ* agonists inhibit release of pro-inflammatory cytokine IL-6 in monocytes.^29^ IL-6 elicits survival response in glioma cells through STAT3.^26^ As heightened STAT3 activation promotes glioma progression, STAT3 inhibitors are regarded as a potential therapeutic target for glioma.^6^ Depletion of pSTAT3 on CIDEA overexpression possibly prevents IL-6 from exhibiting its pro-survival response despite increase in its levels. Decrease in HIF-1*α* transcriptional activation and STAT3 phosphorylation following CIDEA overexpression is concomitant with previous findings that STAT3 is a crucial positive regulator of HIF-1*α* expression.^30^

Dynamics of actin filaments involving their assembly/disassembly and organization influences cell death through an apoptosis-like pathway.^31^ *In silico* analysis predicated that genes associated with cytoskeletal organization are tightly co-expressed with CIDEA in glioblastoma. As de phosphorylated active cofilin is essential for actin cytoskeletal disruption-mediated apoptosis,^32^ it is possible that CIDEA induces apoptosis by altering actin dynamics. Since acetylation of p53 is correlated with apoptotic responses,^22^ CIDEA-induced glioma cell death could also be attributed to increased p53 acetylation. Thus, CIDEA induces glioma cell death by affecting different survival pathways. Taken together, this study not only suggests the existence of a CIDEA-PPAR*γ* regulatory loop, but also demonstrates the role of CIDEA as death inducer in glioma cells. By highlighting the clinical relevance of elevated PPAR*γ* levels in regulating expression of pro-apoptotic CIDEA in glioma, this study warrants further investigation directed towards evaluating efficacy of PPAR*γ* inhibitors as effective anti-glioma therapeutic strategy.

## Materials and Methods

### Processing of patient tissue

Western blot analysis was performed on tissue samples collected from patients with histologically confirmed GBM (*n*=12) to determine CIDEA and PPAR*γ* expression as described.^33^ Non-neoplastic brain tissues obtained from margin of the corresponding tumors were used as control. Samples were obtained as per the guidelines of Institutional Human Ethics Committee of NBRC.

### *In silico* analysis

Data set record number GDS4470 from GEO database was queried for CIDEA, PPAR*γ* and HIF-1*α*. GDS4470 was chosen for its variety of glioblastoma samples from different regions of brain. The expression values for each gene were used to analyze for significant difference by two-tailed Mann–Whitney *U*-test in Sigma Plot version 10.0. Oncomine database was accessed to obtain cancer *versus* normal, and co-expression data in glioblastoma samples. The most significant cancer *versus* normal data was represented. Co-expression data were also obtained from most significant glioblastoma data (TCGA mRNA expression profile) with correlation coefficient of >80%. The co-expressed gene list was then submitted to DAVID Bioinformatics Resources 6.7^34,35^ and significantly enriched pathways were plotted in Microsoft Excel 2007.

### Cell culture and treatment

Human glioma cell lines A172, U87MG and T98G obtained from American Type Culture Collection (Manassas, VA, USA), were cultured in Dulbecco's modified Eagle's medium supplemented with 10% FBS. Semi-confluent cells were transferred to serum-free medium (SFM) for four hours. Cells were then treated with 50 *μ*M of PPAR*γ* inhibitor (T0070907, Tocris Bioscience, Bristol, UK) for 2 h and subsequently co-treated with 20 *μ*M HIF-1*α* inhibitor (LW6, Calbiochem, Billerica, MA, USA) in SFM. DMSO-treated cells served as controls. Glioma cell lines were transfected with CIDEA overexpression plasmid using Lipofectamine 2000 (Life Technologies, Invitrogen, Carlsbad, CA, USA). After 24 h of transfection, cells were treated with 10 *μ*M of JNK inhibitor (SP600125, Tocris Bioscience) in SFM. Following 48 h of treatment, cells were harvested for subsequent analysis. CIDEA overexpression plasmid was a kind gift from Dr. Peter Arner (Karolinska Institute, Stockholm, Sweden).^36^

### Western blot analysis

Western blot analysis was performed with whole lysates, cytosolic and nuclear protein extracts of cells transfected with CIDEA overexpressing plasmid or treated with inhibitors of PPAR*γ*, HIF-1*α* or JNK as described,^33^ using antibodies against CIDEA (Abcam, Cambridge, UK), PPAR*γ*, HIF-1*α* (Novus Biological, Cambridge, UK), phospho-JNK, JNK, phospho-cofilin, Cofilin, phospho-VASP, VASP, phospho-STAT3 (Y705), STAT3, p53, acetyl-p53 (Lys-373 and Lys-382) (Millipore, Billerica, MA, USA); Cyclin B1, *β*-tubulin, GAPDH, C-23 and NF*κ*B Santa Cruz Biotechnology (Santa Cruz, CA, USA). Antibodies were purchased from Cell Signaling (Danvers, MA, USA) unless otherwise mentioned. After addition of horse radish peroxidase-conjugated secondary antibodies (Vector Laboratories Inc., Burlingame, CA, USA) blots were exposed to Chemigenius Bioimaging System (Syngene, Cambridge, UK) and images were developed by Gene snap software (Syngene). Reprobing of the blots was performed after stripping to determine the loading control with anti-*β*-tubulin, GAPDH or C23 antibodies.

### Real-time PCR analysis

Real-time PCR was performed for CIDEA and 18S rRNA using ViiA7 Real Time PCR system (Life Technologies, Invitrogen) as per manufacturer’s protocol with default settings of thermal cycles except for custom annealing temperature for each primer pair. Sequences of the primers were as follows: CIDEA forward: 5′-CCAGCACGTCCCCACTTG-3′, CIDEA reverse: 5′-CGTTAAGGCAGCCGATGAAG-3′, 18S rRNA forward: 5′-CAGCCACCCGAGATTGAGCA-3′, 18S rRNA reverse: 5′-TAGTAGCGACGGGCGGTGTG-3′. Data were analyzed with ViiA7 software.

### Flow cytometric analysis of cell cycle progression and caspase 3 activation

Cells transfected with CIDEA overexpression construct in the presence or absence of JNK inhibitor were harvested and fixed in 1% paraformaldehyde in PBS. The fixed cells were washed in PBS, resuspended in DHE and propidium iodide solution (BD Biosciences, Franklin Lakes, NJ, USA) for 20 min at room temperature and flow cytometric analysis of 10^6^ cells were performed using Cell Quest program on FACS Calibur (Becton Dickinson, San Diego, CA, USA). The percentage of cells in the G1, S and G2/M phases of the cell cycle was analyzed.^37^ Active caspase 3 level was also detected by FACS analysis. In brief, 10^6^ cells were incubated at 4 °C for 40 min with anti-active caspase 3 antibody (Santa Cruz Biotechnology), washed, incubated with anti-rabbit conjugated to FITC secondary antibody for 30 min on ice, washed again, resuspended in PBS and analyzed by FACS.

### Cytokine bead array

Cytokine bead array kit (Human Inflammation CBA kit, BD Biosciences) was used to quantitatively measure cytokine levels in the supernatant collected from CIDEA overexpressing cells treated with or without JNK inhibitor as described.^38^

### Determination of cell viability

Viability of cells treated with PPAR*γ* inhibitor or transfected with CIDEA overexpression plasmid in the presence or absence of 10 *μ*M of JNK inhibitor was assessed using the MTS assay (Promega, Madison, WI, USA) as described.^39^ Values were expressed as fold change over control.

### Luciferase assay

Semi-confluent cells were transfected with 300 ng of CIDEA plasmid, 300 ng of the HIF-1*α* responsive element luciferase construct (a kind gift from Chinmay Mukhopadhyay, JNU, India ) and 10 ng of Renilla luciferase vector (pRL-TK, Promega, as transfection control) using Lipofectamine 2000 (Life Technologies, Invitrogen). After 48 h, cells were harvested and luciferase activity was measured using the Dual-Luciferase Reporter Assay System Kit (Promega) according to manufacture protocol in a GloMax 96 microplate luminometer.^40^

### Immuno-cytochemistry

Immuno-cytochemistry was performed to determine actin cytoskeleton architecture in glioma cells transfected with CIDEA overexpression plasmid in the presence and absence of JNK inhibitor. Following 40 h of treatment, cells were fixed with 4% formaldehyde. Fixed cells were then incubated in PBS containing 1% BSA for 30 min, followed by incubation with Cofilin antibody at 4 °C in staining solution (containing 6.25 *μ*l rhodamine labeled phalloidin, 2.5 mg BSA in 250 *μ*l PBS) overnight at 4 °C. Cells were washed and labeled with Alexa fluor 488 secondary antibody, washed, mounted with Vectashield mounting medium with DAPI (Vector Labs, Inc.). CIDEA regulated localization of JNK was determined using Mitotracker green and JNK antibody followed by subsequent incubation with Alexa fluor 594 labeled secondary antibody. Cells were washed with PBS, mounted and immunofluorescence was recorded using Apotome upright fluorescence microscope (Carl Zeiss) as described previously.^41^

### ChIP and ChIP real-time PCR assays

ChIP was performed on glioma cells treated with PPAR*γ* inhibitor for 48 h by Chip-IT Enzymatic DNA shearing Kit (Active Motif, Carlsbad, CA, USA) as described previously.^42^ Following treatment, cells were fixed with 1% formaldehyde at room temperature for exactly 8 min, and further processed according to manufacturer’s instruction. Anti-NF*κ*B (Santa Cruz Biotechnology) and anti-SP1 (Cell signaling) were used for immunoprecipitation and non-specific IgG antibody (Abcam) was used as control. After reverse cross-linking and DNA purification, DNA from input (1:10 diluted) or immune-precipitated (IP) samples were quantified by real-time PCR using ABI 7500 real-time thermal cycler with Power SYBR green PCR Master Mix (Life Technologies, Invitrogen) for 40 cycles. Threshold cycle number (Ct) values of IP samples were normalized by corresponding Ct of 1% input DNA. The relative fold change value was analyzed relative to the control. Primer sequences of CIDEA promoter region used for ChIP real-time PCR analysis are listed in Supplementary Table 1.

## Abbreviations

CIDEA: cell death-inducing DFFA-like effector A
PPAR gamma: peroxisome proliferator-activated receptor gamma
HIF-1 alpha: hypoxia inducible factor 1 alpha
GAPDH: glyceraldehyde-3-phosphate dehydrogenase
NF*κ*B: nuclear factor kappa B
Sp1: specificity protein 1
c23: nucleolin
VASP: vasodilator-stimulated phosphoprotein
STAT3: signal transducer and activator of transcription 3
JNK: c-Jun *N*-terminal kinase
GBM: glioblastoma multiforme
IL-6: interleukin-6
TSS: translation start site
CID/CIDEA OE: CIDEA overexpression
T007: PPAR gamma inhibitor, commercial name-T0070907
mg: milligram
*μ*g: microgram
mM: milimolar
*μ*M: micromolar
hrs: hours.
