# Supplementary material for: PPARγ regulated CIDEA affects pro-apoptotic responses in glioblastoma
Source: Cell Death Discov. 2015 Nov 23;1:15038–. doi: 10.1038/cddiscovery.2015.38 (PMC4979534; doi:10.1038/cddiscovery.2015.38)
Supplement: Supplementary Table 1 [file cddiscovery201538-s1.doc]

| **Sequence (5′-3′)** | **Base pair position of amplified region** |
| --- | --- |
| CTGTAGTGAGGGGTTCTGGG | -839 to -737 |
| CCTGCTCTCATGGTTGTTCC |
| AGTGACCAAAAGAGACCCGG | -266 to -93 |
| GCAGGGGAGGACGTTTTG |
| CAGGCAGACAGACCTCCAG | -26 to +120 |
| GAATATGAGGCCACCGAACG |

**Supplementary Table-1:** Primer sequences for amplification of NFkB and SP1 binding on regulatory genomic region of CIDEA
