# Supplementary figures and images for: PPARγ regulated CIDEA affects pro-apoptotic responses in glioblastoma
Source: Cell Death Discov. 2015 Nov 23;1:15038–. doi: 10.1038/cddiscovery.2015.38 (PMC4979534; doi:10.1038/cddiscovery.2015.38)

Supplementary Figure 1

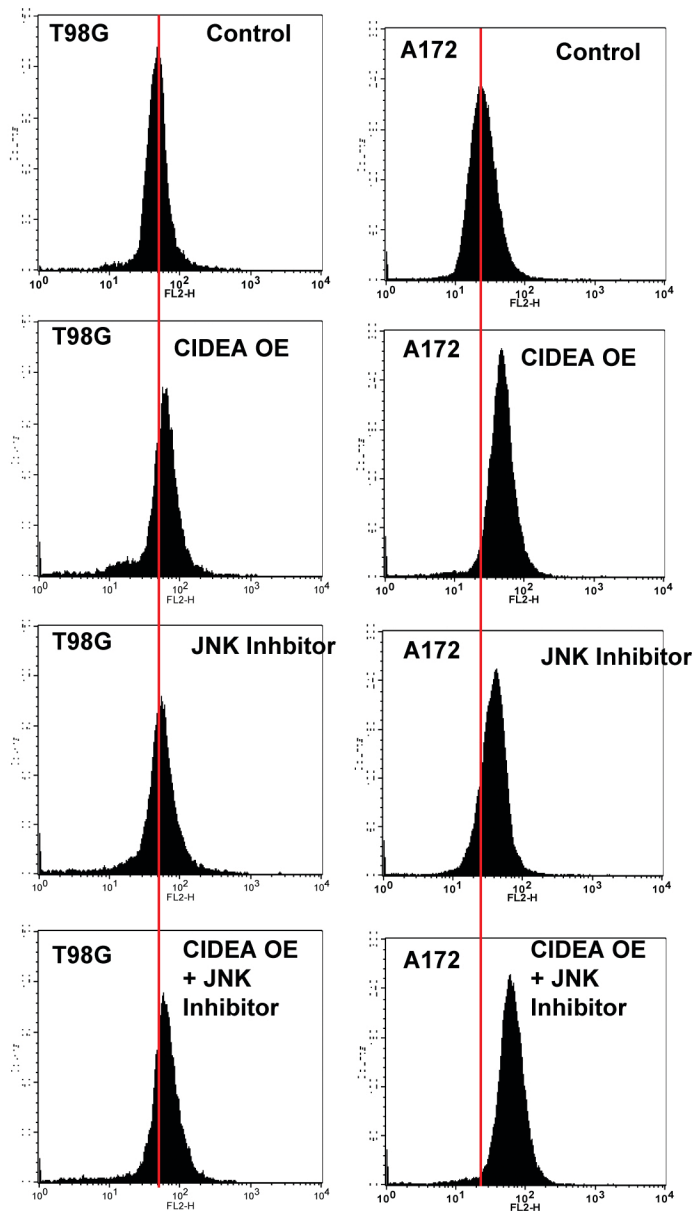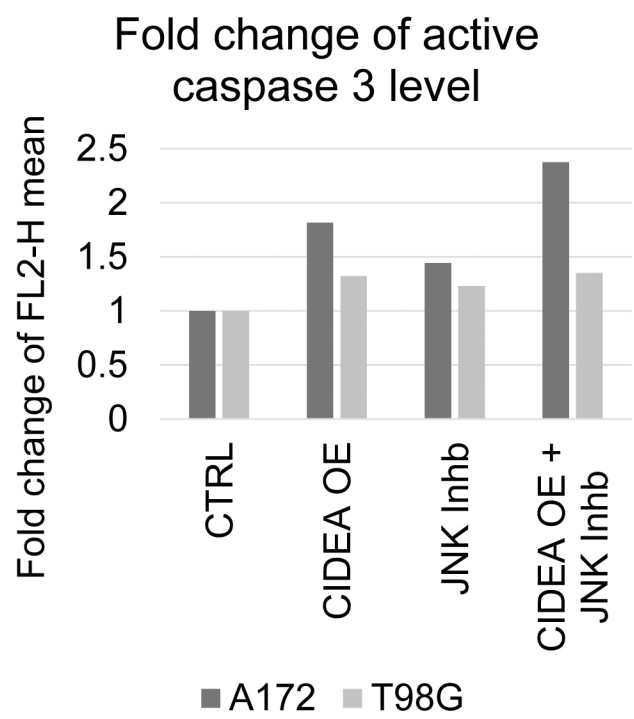

Supplementary Figure 2

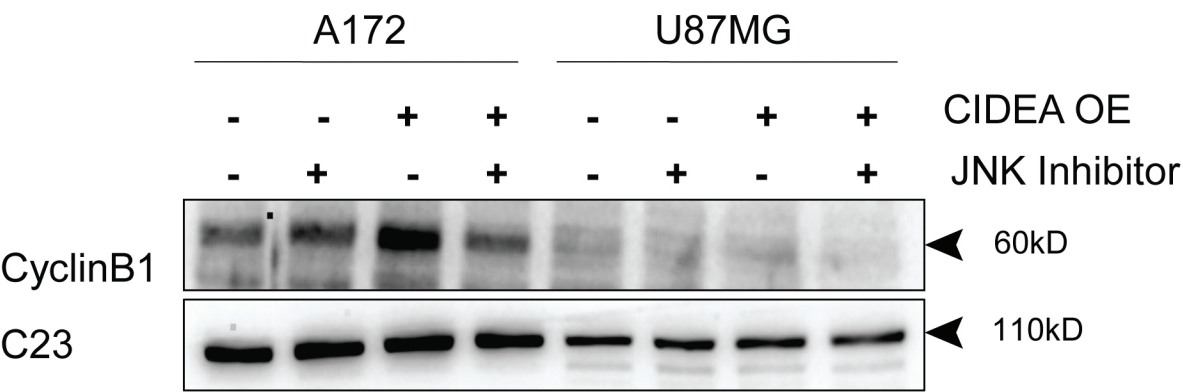

Supplementary Figure 3

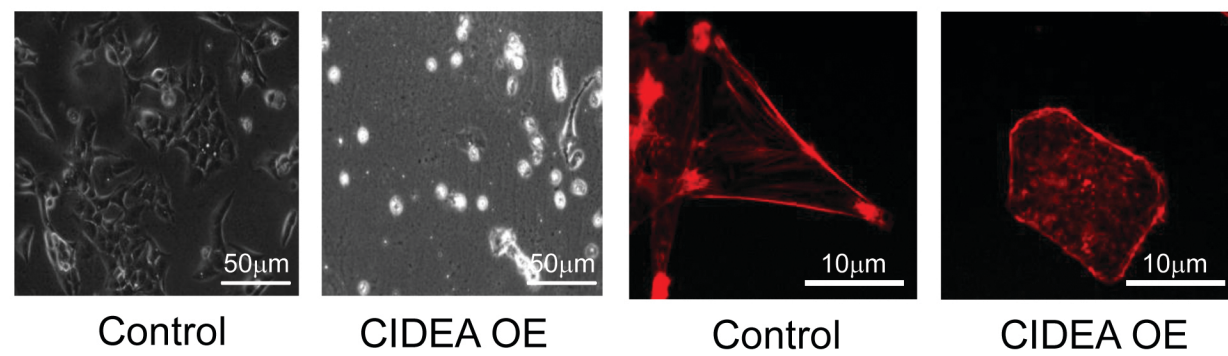

Supplement: Supplementary Figures [file cddiscovery201538-s2.pdf]
